# Edge Effects on the Spatial Distribution and Diversity of Drosophilidae (Diptera) Assemblages in Deciduous Forests of Central European Russia

**DOI:** 10.3390/insects16080762

**Published:** 2025-07-24

**Authors:** Nikolai G. Gornostaev, Alexander B. Ruchin, Oleg E. Lazebny, Alex M. Kulikov, Mikhail N. Esin

**Affiliations:** 1 N.K. Koltzov Institute of Developmental Biology, Russian Academy of Sciences (RAS), 119334 Moscow, Russia; n_gornostaev@mail.ru (N.G.G.); o.e.lazebny@idbras.ru (O.E.L.); amkulikov@gmail.com (A.M.K.); 2 Joint Directorate of the Mordovia State Nature Reserve and National Park «Smolny», 430005 Saransk, Russia; esinmishka@gmail.com

**Keywords:** Diptera, dynamics, abundance, species diversity, insects, Republic of Mordovia

## Abstract

In the forest ecosystems of Central European Russia, the influence of forest edges on the spatial distribution of Drosophilidae was studied for the first time. The research was conducted during the period of 2021–2022 in the Republic of Mordovia. Beer traps baited with fermented beer and sugar were used to collect Drosophilidae. Two study plots were selected, differing in their forest edges, tree stands, and adjacent open ecosystems. In both cases, the forest directly bordered an open ecosystem. Edges serve as transitional biotopes, where both forest and meadow (open area) faunas coexist. Knowing that many drosophilid species prefer forest habitats, we designated forest interior sites as control points. Traps were set at heights of 1.5 m (lower) and 7.5 m (upper) on trees. A total of 936 specimens representing 27 species were collected. Nine species were common across all traps, while ten species were recorded only once. At the forest edges, 23 species were captured across both heights, compared to 19 species in the forest interiors. However, the total abundance at the forest edges was 370 specimens, while it was 1.5 times higher in the forest interiors. Both abundance and species richness varied between plots. Margalef’s index was higher at the forest edges than in the forest interiors, particularly at 1.5 m height at the edge and at 7.5 m height in the forest interior. Shannon and Simpson indices showed minimal variation across traps at different horizontal and vertical positions. The highest species diversity was observed among xylosaprobionts (9 species) and mycetophages (8 species). All ecological groups were represented at the forest edges, whereas only four groups were recorded in the forest interiors, with the phytosaprophagous species *Scaptomyza pallida* being absent. In general, both species richness and drosophilid abundance increased in the lower strata, both at the forest edge and within the interior. Using the R package Indicspecies, we identified *Gitona distigma* as an indicator species for the forest edge and *Scaptodrosophila rufifrons* as an indicator for the forest interior in the lower tier for both plots. In addition, *Drosophila testacea*, *D. phalerata*, and *Phortica semivirgo* were found to be indicator species for the lower tier in both plots, while *Leucophenga quinquemaculata* was identified as an indicator species for the upper tier at the second plot.

## Simple Summary

This study investigates how the edges of forests affect the types and numbers of small flies known as Drosophilidae in Central European Russia. These flies live in different parts of the forest and nearby open areas like meadows. Researchers set up traps at two forest locations to catch these flies at two heights on trees. They found 27 different species, with some species living mostly at the forest edges and others more inside the forest. While more flies were caught inside the forest, there were more species variety at the edges. The variety of flies also changed depending on how high the traps were placed. Certain fly species were found only at the edges or only inside the forest, which helps us understand which species prefer specific habitats. These findings are important for understanding how forest borders influence insect communities, which can affect the health of forest ecosystems and biodiversity. Knowing how insects respond to changes in forest environments can help in conservation and ecological management.

## 1. Introduction

The family Drosophilidae is among the most ecologically diverse groups within the order Diptera, encompassing over 4600 species across 76 genera globally, including more than 120 species recorded in Europe. The genus *Drosophila* is the largest within the family [1]. Members of this family inhabit a wide range of natural and anthropogenic environments. A significant portion of drosophilid species is typically associated with forest ecosystems, although many have successfully adapted to human-modified habitats [2,3,4]. Numerous species are linked to ripe fruits and fungi and are attracted to decaying or fermenting organic substrates [5,6]. Their larvae develop in a wide variety of decomposing substrates, feeding on the microorganisms inhabiting these environments. These substrates include rotting vegetables, fruits, fungi, plant debris, sap fluxes decaying wood and even parasitism, kleptoparasitism, entomophagy [7,8,9,10]. Notably, *D. suzukii*, one of the few true agricultural pests in the family, has spread globally, and causes significant damage to soft fruits. It infests a variety of hosts plants such as *Prunus* sp., *Rubus* sp., *Fragaria* sp. (Rosaceae), *Vaccinium* sp. (Ericaceae), and *Vitis* sp. (Vitaceae) [11]. Some drosophilid larvae are capable of developing in substrates with high ethanol concentrations [12], while others are known as leaf and stem miners on economically important crops, including legumes, chenopods and brassicas [13,14,15]. In tropical regions, many drosophilid species breed in flowers, sometimes specializing on a single flowering plant species [7,10]. Drosophilidae are distributed worldwide and are remarkably adaptable, aided by high reproductive rates and strong dispersal capabilities. These traits make some species difficult to manage in agricultural systems, particularly *D. suzukii*, recognized as a major global pest of soft fruits [15]. At the same time, several drosophilid species are sensitive to anthropogenic disturbances and serve as valuable bioindicators for assessing habitat degradation. Others, such as *D. suzukii*, *D. ananassae*, *D. busckii*, *D. immigrans*, *D. melanogaster*, and *D. simulans* are known to invade disturbed and high-stress environments [16].

Ecotones are transitional zones between ecological communities, typically formed along environmental gradients. These gradients arise from spatial variations in local plant communities and microhabitats [17]. In sufficiently large ecosystems, forest edges represent pronounced ecotones—abrupt boundaries between two relatively homogeneous habitats. These zones separate closed forest systems from open landscapes such as meadows, pastures, or clearings. Under such conditions, edge effects are commonly observed, manifesting as alterations in various environmental parameters [18,19]. Studies investigating the influence of edge effects on specific insect groups or individual species have yielded divers outcomes. In some instances, the effect appears minimal, while in others, it penetrates into the forest interior to varying extents [20,21,22,23,24,25,26]. These discrepancies may be attributed to a wide range of factors, some of which are not always accounted for in ecological assessments.

In Brazil, species such as *D. simulans*, *S. latifasciaeformis*, and *Z. indianus* have been predominantly collected at forest edges, whereas *D. willistoni* and *D. mediostriata* were more frequently found in forest interiors [27]. Seasonal differences in the abundance and diversity of Drosophilidae between edge and interior habitats have also been reported in Brazil [18]. In Switzerland, most drosophilid species exhibited greater abundance at forest edges compared to the forest interior [28].

The aim of this study was to assess, for the first time in central European Russia, the influence of forest edges on the abundance and species diversity of Drosophilidae. The specific objectives were (1) to compare the number of species and individuals at forest edges and in forest interiors; and (2) to evaluate species richness and abundance at two different vertical strata above the ground.

## 2. Materials and Methods

**Figure 1:**
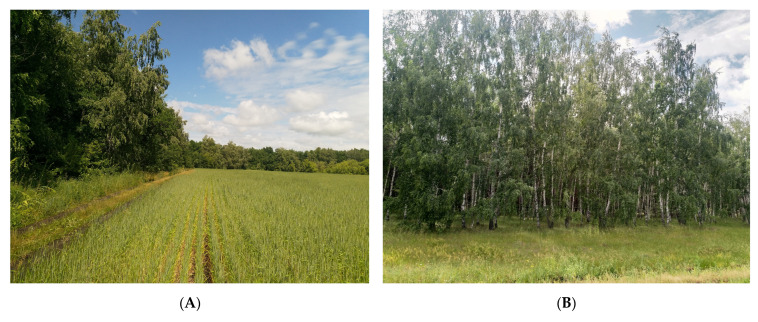
Photographs of the study habitats: (**A**)—plot 1; (**B**)—plot 2.

The study was conducted in the Republic of Mordovia, located in the central part of European Russia (Figure 1). Research plots were situated within forest ecosystems of the Oka-Don Plain. Two study sites were selected, each comprising a deciduous forest adjacent to an open ecosystem (either a meadow or regularly ploughed field). The primary distinction between the two plots was the type of adjacent open habitat. However, the forest ecosystems themselves also exhibited certain differences, described in more detail below. The area of each forest fragment ranged from 0.6 to 0.8 hectares. The two plots were located approximately 30 km apart.

At the boundary between the forest and the adjacent open area, a forest edge was formed. Within the forest, a control area—referred to as the forest interior—was designated 300–350 m from the edge and characterized by a closed canopy. Each plot contained eight traps placed in four positions: edge–lower, edge–upper, interior–lower, interior–upper, with two traps installed per position. Traps were suspended on tree branches at two vertical strata: 1.5 m (lower) and 7.5 m (upper) above the ground.

These specific heights were chosen based on findings from our recent study on the vertical distribution of drosophilids in the Mordovian State Nature Reserve [29], which revealed differences in both species richness and abundance across vertical forest layers. The 1.5 m height corresponds to the forest’s lower tier, which is typically inhabited by mycophagous drosophilids associated with basidiomycete fungi. Xylosaprobiont species occur at various heights, depending on the availability of trunk wounds sap flows. The canopy layer is also favored by the drosophilid *Leucophenga quinquemaculata*, whose larvae develop in tinder fungi growing on tree trunks.

At the forest edge of Plot 1 (54.4817° N, 43.5201° E), the upper canopy was dominated by *Quercus robur*. The lower canopy layer included *Pinus sylvestris* and *Betula pendula*. The shrub layer was dense, with a projective cover of 30%, and was primarily composed of *Sorbus aucuparia*, juvenile *Quercus robur*, and *Malus domestica*. The herbaceous-shrub layer, with up to 70% cover, comprised *Fragaria vesca*, *Galium mollugo*, *Filipendula ulmaria*, *Pimpinella saxifraga*, *Hypericum perforatum*, *Viola hirta*, *Campanula trachelium*, *Campanula rapunculoides*, *Glechoma hederacea*, *Agrimonia eupatoria*, *Poa nemorosa*, *Galium boreale*, *Aegopodium podagraria*, *Geranium sylvestre*, *Dactylis glomerata*, *Veronica chamaedrys*, *Vicia sepium*, and *Geum urbanum*.

In the forest interior, the upper canopy had a projective cover of 80% dominated by *Quercus robur* and *Betula pendula*. The second layer was composed solely of *Betula pendula*. The shrub layer, with 50% cover, included *Sorbus aucuparia*, *Prunus padus*, *Amelanchier spicata*, *Frangula alnus*, *Viburnum opulus*, *Sambucus racemosa*, and juvenile *Quercus robur*. The herbaceous-shrub layer, 80% cover, comprised *Stellaria holostea*, *Aegopodium podagraria*, *Glechoma hederacea*, *Lathyrus vernus*, *Geum urbanum*, *Campanula rapunculoides*, *Betonica officinalis*, *Pimpinella saxifraga*, *Viola hirta*, *Agrimonia eupatoria*, *Carex spicata*, *Calamagrostis arundinacea*, and *Scrophularia nodosa*. Adjacent to this forest was an agroecosystem where winter wheat (*Triticum aestivum*) was cultivated.

At the forest edge of Plot 2 (54.7270° N, 43.3272° E), the upper canopy consisted exclusively of *Betula pendula*, with a projective cover of 20–30%. The secondary (shrub) layer was sparse and included *Salix caprea*, *S. cinerea*, *Frangula alnus*, *Malus domestica*, and *Sorbus aucuparia*. Both canopy layers were highly transparent. The herbaceous layer was well-developed (80–85% cover) and included species such as *Seseli libanotis*, *Veronica chamaedrys*, *Phleum pratense*, *Melampyrum nemorosum*, *Leucanthemum vulgare*, *Trifolium hybridum*, *Pimpinella saxifraga*, *Knautia arvensis*, *Achillea millefolium*, *Hypericum perforatum*, *Vicia cracca*, *Linaria vulgaris*, *Erigeron annuus*, *Cichorium intybus*, *Deschampsia cespitosa*, *Angelica sylvestris*, *Lysimachia vulgaris*, *Anthoxanthum odoratum*, and *Galium mollugo*.

In the forest interior, the canopy was completely composed of *Betula pendula*. The shrub layer was even sparser (projective cover 5–10%) and included *Frangula alnus*, *Sorbus aucuparia*, and small trees such as *Populus tremula*. The herbaceous layer consisted of *Fragaria vesca*, *Pimpinella saxifraga*, *Platanthera bifolia*, *Rubus saxatilis*, *Melampyrum nemorosum*, *Viola canina*, *Phleum pratense*, *Plantago lanceolata*, *Hypericum perforatum*, *Galium mollugo*, *Dryopteris carthusiana*, *D. expansa*, *Leucanthemum vulgare*, *Agrostis gigantea*, *Convallaria majalis*, *Deschampsia cespitosa*, *Chamaenerion angustifolium*, and *Calamagrostis epigeios*. This deciduous forest bordered a meadow ecosystem devoid of trees and shrubs. The herbaceous vegetation of the meadow was dense and composed of typical meadow grasses and a variety of composite plant species.

*Drosophilidae* samples were collected during June and July during the years 2021–2022. These months were selected based on our previous research on the seasonal dynamics of drosophilid abundance and species diversity in Mordovia [29,30]. In this region, located in the forest zone of Central European Russia, both drosophilid abundance and species richness peak during the summer months. In contrast, May typically shows noticeably lower numbers, likely due to variation in the timing of emergence from winter diapause, which may occur at different life stages (pupa or imago). In June and July, drosophilid populations’ density and diversity increase significantly. However, by August, a marked decline is observed, possibly associated with generational turnover and preparation for diapause in some species. In autumn (September–October), unstable weather conditions—characterized by wind, rainfall, and frost—can strongly affect fly abundance, especially at forest edges exposed to open habitats. Therefore, June and July represent the optimal period for studying edge effects in Mordovia.

Drosophilids were collected using beer traps made from 5 L plastic containers, each with a single cut-out window to allow insect entry [31]. The bait consisted of beer mixed with sugar, a method we have successfully employed for many years in fruit fly studies. Trapping was conducted continuously over a two-month period, with samples collected every 15–17 days (three sampling events per season). In the laboratory, all insects were washed and sorted under a stereomicroscope by order and family. Drosophilidae specimens were preserved in plastic vials containing 70% ethanol. Species identification and counting were subsequently performed. The analysis was based on total drosophilid abundance in all traps. Biodiversity metrics were calculated using standard indices, including the Margalef index, Shannon index, and Simpson index [32,33].

Estimates of the mean, median, standard deviation, and quartiles of species abundance in the samples were calculated. The distribution of species abundance values was tested for normality using the Kolmogorov–Smirnov and Lilliefors tests (Table S1).

To test the hypothesis of random differences in species composition across samples differing by vertical stratum, forest edge/interior position, and plot, a G-test was employed for comparing the distribution of individual counts across all species [34]. Calculations were performed in Excel. To assess the significance of differences in the proportion of each species across summary datasets differing by vertical stratum, a position relative to the forest edge, and a sampling plot, 2 × 2 contingency tables were used with the χ^2^ test implemented in Statistica 12 (StatSoft, Inc., Tulsa, OK, USA). Given the multiple comparisons, the Benjamini–Hochberg correction was applied to control the false discovery rate (FDR).

Species distributions expressed as their proportional contribution to the total abundance in each trap are visualized in diagrams generated using Excel. Data were grouped by similarity in one of the following factors: vertical stratum (trap height), forest position (edge vs. interior), and sampling plot. Species with fewer than four individuals in total were excluded from visualization.

To compare drosophilid species distributions across communities while simultaneously accounting for vertical stratum, forest position, and plot, cluster analysis was performed using Statistica 12 (StatSoft, Inc.). A distance matrix was computed from Spearman rank correlation coefficients between all pairs of samples, and the weighted pair-group average method (WPGMA) was used as the clustering algorithm. This method was selected because of the significant deviation of the abundance distributions from normality and the unbalanced total counts across samples. WPGMA is well suited for datasets containing both independent cluster groups and chain-like structures, as well as for those with large differences in cluster sizes.

To more comprehensively assess the statistical relationship between species abundance and trap location, and to identify indicator drosophilid species, the R package Indicspecies (https://www.R-project.org) was used [35,36]. The clustering of sampling plots was performed independently for each variable—vertical stratum, forest position and sampling plot. The function multipatt (multi-level pattern analysis) was applied to identify species reliably associated with specific groups of plots using the IndVal (Indicator Value) index. The combine species function was used to create a species-by-site-group matrix, and the multipatt function was again used to assess the strength of association between species combinations and plot groups.

## 3. Results

A total of 936 Drosophilidae specimens were examined. Across all sites, 27 species belonging to 10 genera were collected and identified (Table 1). At the forest edges, 23 species were collected at two different vertical levels, whereas only 19 species were found within the forest interior. However, the overall abundance was 370 specimens at the forest edges, while the abundance in the forest interior was 1.5 times higher.

Nine species were identified across all traps: *A. alboguttata*, *A. rufescens*, *G. distigma*, *L. quinquemaculata*, *D. obscura*, *D. phalerata*, *D. testacea*, *D. transversa*, and *S. rufifrons*. Ten species were represented by single specimens and were found in only one trap: *A. albilabris*, *A. subtusriata*, *S. coleoptrata*, *S. furta*, *D. hydei*, *D. littoralis*, *D. melanogaster*, *D. repleta*, *H. trivittata*, and *S. pallida*.

The highest Margalef index values were recorded at the forest edge at 1.5 m height and within the forest at 7.5 m (Table 1). Overall, this index was higher at the forest edge than inside the forest. The Shannon and Simpson indices varied by approximately 10% across traps located at different spatial and vertical positions and showed no significant differences. These indices were nearly identical for the combined data from the forest edge and interior.

**Figure 2:**
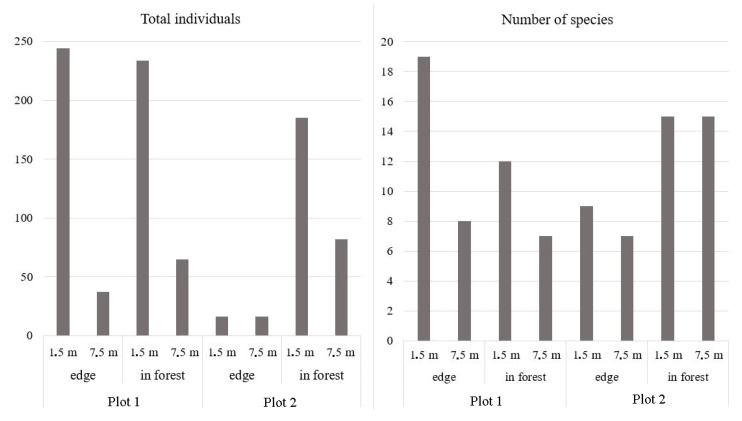
Total abundance and species richness of Drosophilidae collected using beer traps at different plots and heights.

In plot 1, 21 species were recorded. At the forest edge, 19 species were collected (281 specimens), whereas only 12 species were found within the forest (299 specimens). The highest species diversity and abundance in plot 1 were observed at a height of 1.5 m, both at the forest edge and inside the forest (Figure 2). The most abundant species was *D. obscura*, which is typical for the central European part of Russia. *D. testacea*, *D. transversa*, and *D. phalerata* were less abundant. The remaining species were represented by fewer than 10 individuals.

In plot 2, a total of 19 species were recorded. At the forest edge, 12 species were collected, with a total abundance of 32 specimens. Inside the forest, 17 species were identified, with a total abundance of 267 specimens. At the forest edge, *Drosophilidae* abundance was identical at both trap heights and the number of species was nearly the same. Within the forest, the species count did not differ between the heights, but the number of specimens at 1.5 m was 2.3 times higher than in the upper traps. As in plot 1, *D. obscura* was the most abundant species in plot 2. Additionally, four other species—*L. quinquemaculata*, *D. histrio*, *D. testacea*, and *S. rufifrons*—showed comparable abundance levels, each ranging from 30 to 38 specimens.

**Figure 3:**
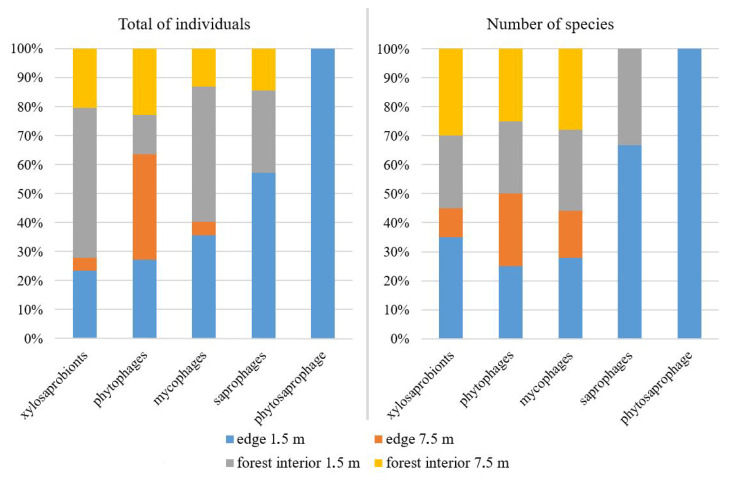
Total abundance and species richness of different ecological groups of *Drosophilidae* collected using beer traps at various plots and heights.

Based on the analysis of ecological groupings, 24 species from five groups—xylosaprobionts, mycophages, phytosaprophages, phytophages, and saprophages—were identified (Figure 3). Only three species (*A. alboguttata*, *A. rufescens*, and *A. subtusradiata*) were not assigned to any ecological group. The highest species richness was observed among xylosaprobionts (nine species) and mycophages (eight species). In terms of abundance, mycophages dominated. All ecological groups were present at the forest edges, while only four groups were found inside the forest—the phytosaprophage *S. pallida* was absent. In general, both the species richness and the abundance of drosophilids increased in the lower tier at both the forest edge and the forest interior.

The G-test was used to assess the effects of all three factors—forest tier, forest depth, and site—on Drosophilidae abundance in the samples. This test accounts for the polynomial distribution of categories in the compared samples, and a significant result indicates substantial differences in the distribution of the total abundance of individuals in accordance to the factor. The summarized data demonstrate a significant effect of all three factors—forest tier, forest depth, and plot—on the species composition in the samples (Table 2).

**Figure 4:**
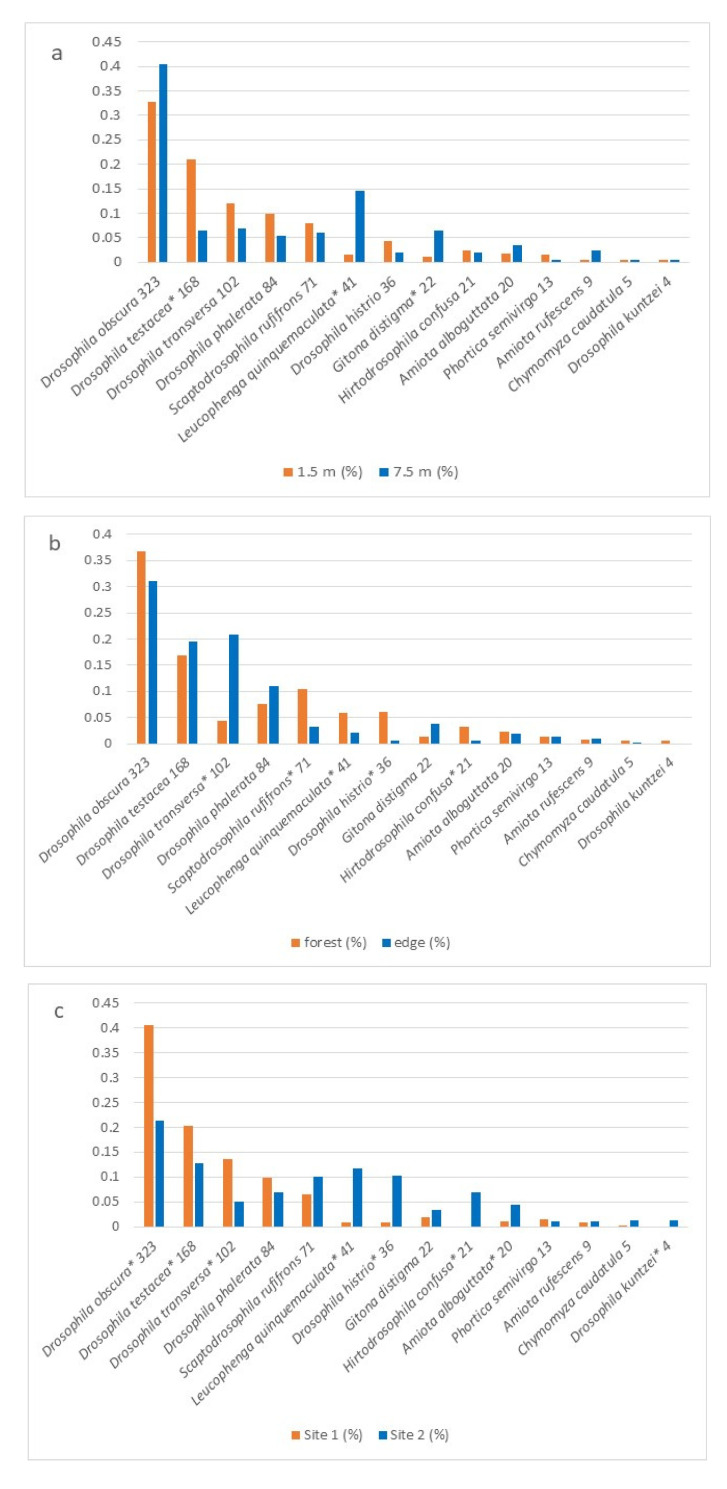
Proportion of each species in the collections depending on the forest layer (**a**), trap location relative to the forest edge (**b**), and plot (**c**). The total number of individuals collected for each species is indicated after the species name. Species represented by fewer than four individuals in the collections are not shown in the diagrams. Asterisks * indicate species with statistically significant differences in relative abundance between compared groups.

The influence of all three factors on Drosophilidae in our collections is presented in Table 3. Taking multiple comparisons into account, collections from the lower and upper forest layers differ significantly for three drosophilid species: *G. distigma*, *L. quinquemaculata*, and *D. testacea* (Figure 4a). Thus, the abundant species *D. testacea* is characteristic of the lower forest layer, whereas *G. distigma* and *L. quinquemaculata* are predominantly found in the upper layers. The upper layer is also characterized by the rare species *A. albilabris*, which was not found in the lower layer collections. The position of traps at the forest edge vs. the forest interior significantly affected five species: *L. quinquemaculata*, *D. histrio*, *D. transversa*, *H. confusa*, and *S. rufifrons*. Among them, only *D. transversa* dominated in the forest edge collections, while the four remaining species were more abundant in the forest interior (Figure 4b). Additionally, significant differences were observed between the Drosophilidae communities of Plot 1 and Plot 2. The most numerous species in the collections—*D. obscura*, *D. testacea*, and *D. transversa*—dominated in Plot 1, while species with moderate or low abundance—*L. quinquemaculata*, *D. histrio*, *H. confusa*, *A. alboguttata*, and *D. kuntzei*—were more typical of Plot 2 (Figure 4c).

Interestingly, a significant dependence on two or all three factors was observed in the distribution of five species: *L. quinquemaculata*, *D. testacea*, *D. transversa*, *D. histrio*, and *H. confusa*. For example, *L. quinquemaculata* was characteristic of the collections from the upper forest layer in the deep forest of Plot 2, while *D. transversa* was typical for samples from both forest layers at the edge of Plot 1. On the other hand, four species with high or moderate abundance—*G. distigma*, *S. rufifrons*, *D. obscura*, and *A. alboguttata*—showed significant dependence on only one factor.

**Figure 5:**
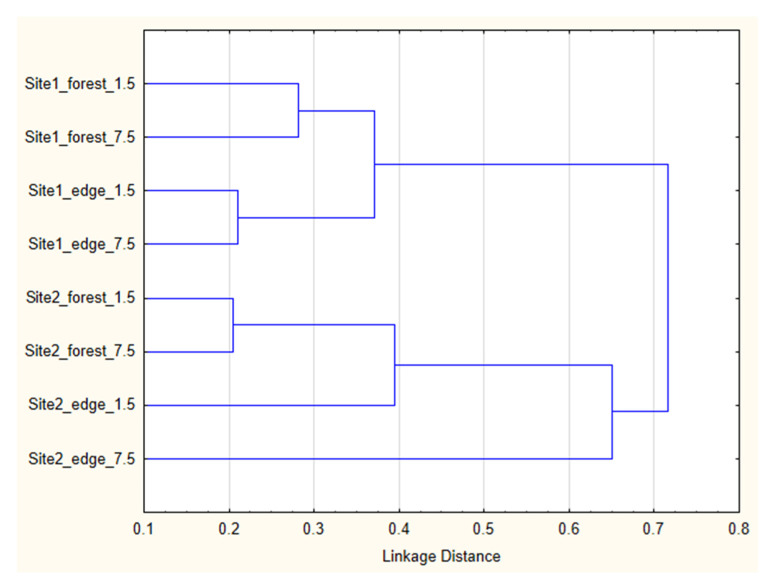
Cluster analysis of drosophilid communities based on the similarity of their species composition. Linkage rule: Weighted pair-group average; Distance measure: Spearman rank order correlations.

To further analyze the overall pattern of similarity in species composition among samples from different biotopes, cluster analysis was performed (Figure 5). The resulting distribution of samples across various habitats largely reflects the conclusions drawn regarding the influence of ecological factors on the drosophilid community composition. The greatest number of species showed a significant dependence on “plot”. Among the eight such species, three were highly abundant, which considerably increased the Spearman rank correlation values, and, consequently, reduced the distance between related clusters. Still samples from Plot 1 and Plot 2 formed two distinct clusters (Figure 5). Five species showing dependence on the “forest depth” factor included one abundant species and four species with moderate abundance. These species contributed to the second level of clustering, associated with the location of traps at the forest edge or interior. The influence of forest layer (upper vs. lower) was significant for four species and represented the third most important factor. Nevertheless, the division of “forest” and “edge” clusters into subclusters corresponding to the upper and lower layers consistently indicated substantial differences in species composition and abundance of drosophilids. For instance, in the edge community of Plot 2, the effect of forest layer was notably stronger than that of the forest depth, largely due to the influence of *L. quinquemaculata*.

A key limitation of the standard statistical assessments of differences in biotopes based on species composition lies in the probabilistic nature of abundance estimates for each species in the samples. These estimates do not allow for the definitive identification of a species as an indicator, since the observed differences do not exclude the species’ presence in alternative biotopes. To independently verify our conclusions regarding the statistical significance of species–site associations, and to more rigorously identify indicator species, we performed an analysis of species abundance distributions in the samples using the R package Indicspecies. The function multipatt was used to determine the significance of individual species as indicators for specific biotopes (Table 4).

In the present study, we grouped the samples into two clusters in three different ways using the factors “forest layer,” “forest depth,” and Plot 1 and 2. For the large cluster defined by the “lower forest layer” factor, several indicator species were identified—*D. testacea*, *D. phalerata*, and *P. semivirgo*. Two species, *L. quinquemaculata* and *H. confusa*, were found to be characteristic of Plot 2, with *L. quinquemaculata* serving as an indicator exclusively for the upper layer of this plot. For the drosophilid communities from the forest interior of Plot 2, the species *D. kuntzei*, *H. confusa*, and *C. caudatula* were typical. *G. distigma* was an indicator species for drosophilid communities inhabiting the forest edge. *S. rufifrons* was characteristic of drosophilid communities residing in the lower forest layer deep inside the forest.

Indicator taxa for habitats may include not only single species but also their combinations. An analysis of such combinations, consisting of two or three drosophilid species, revealed several possible species–indicator sets for different forest layers in Plot 1 and for the lower layer of both plots (Table 5).

For Plot 1, indicator combinations may include two species—*D. phalerata* and *D. testacea*, or three species—*D. obscura*, *D. phalerata*, and *D. testacea*. For the lower forest layer in both forest interior and edge habitats across the two plots, 14 possible species pairs were identified, predominantly involving the combinations of 5 species: *D. testacea*, *D. transversa*, *D. phalerata*, *D. obscura*, and *P. semivirgo*. *S. rufifrons* was occasionally included in these combinations (Table 5).

## 4. Discussion

This study provides the first insights into the species diversity and abundance of Drosophilidae at the forest edges of temperate deciduous forests in European Russia. Forest edges are ecotonal habitats where both forest-dwelling and open-area (meadow) faunal elements may coexist. Given that many drosophilid species are known to prefer forest habitats, we used forest interior sites as reference (control) locations. Previous studies have demonstrated that various Diptera families and species exhibit distinct responses to forest edges. For example, certain Culicidae species have been found in higher abundance deeper within forests, with significantly lower numbers recorded near forest edges [37]. Similarly, *Anopheles* mosquitoes were reported to be more prevalent in forest interiors, with 66.3% of individuals collected there compared to only 33.7% at the edges [38]. In contrast, in forest ecosystems in Argentina, the species richness and abundance of Diptera were higher at the forest edge than within forest fragments [39]. Comparable patterns have been observed in anthropogenic landscapes. For instance, more species and individuals of Syrphidae were captured at the edges of apple orchards than in their interior zones [40]. As noted previously, certain Drosophilidae species also exhibit a preference for forest edges in various forest ecosystems [16,27,28].

In our study, the most abundant species (with more than 10 specimens in total) were *A. alboguttata*, *G. distigma*, *L. quinquemaculata*, *P. semivirgo*, *D. histrio*, *D. obscura*, *D. phalerata*, *D. testacea*, *D. transversa*, *H. confusa*, and *S. rufifrons*. Among these, only two species showed a marked preference for the forest edge over the interior: *G. distigma* (14 specimens at the edge vs. 8 in the interior) and *D. transversa* (57 specimens at the edge vs. 25 in the interior). The larvae of *G*. *distigma* are phytophagous and develop in the inflorescences of various herbaceous plants in the Asteraceae family, such as *Sonchus* and *Crepis*, which are more abundant at forest edges and in adjacent meadow habitats [41,42]. *D. transversa* larvae are typical mycophages, feeding on various species of basidiomycete fungi [43], many of which grow in greater abundance along forest edges, likely due to higher humidity and greater light availability. Another mycophagous species, *D. phalerata* [43], was collected in nearly equal numbers at the forest edge (37 specimens) and in the forest interior (43 specimens), suggesting a broader ecological tolerance.

The eight remaining abundant Drosophilidae species exhibit a clear preference for the forest interior, although the strength of this preference varied among species. The most frequently encountered species in our collections was *D. obscura*. This species is widespread throughout Europe [44,45] and has previously been reported as the most numerous drosophilid in forest ecosystems of the temperate zone in Russia [29,30,46]. *D. obscura* belongs to the ecological group of xylosaprobionts, with larvae developing under the bark, in moist wood tissues, and in exuding sap [47]. In our study, the abundance of *D. obscura* was significantly higher in lower traps than in those placed in the tree canopy, both at the forest edge and within the interior (222 specimens in lower traps vs. 81 in canopy traps). This indicates a marked preference for the forest floor or understory microhabitats. Interestingly, this vertical distribution contrasts with findings from Swiss forests, where *D. obscura* was more frequently recorded in the canopy [28].

Among the abundant species in our collections, the group of xylosaprobionts also includes *P. semivirgo* and *S. rufifrons* [42,48,49]. These species exhibited a similarly uneven vertical distribution, showing a marked preference for the lower forest stratum. This pattern is likely associated with the higher concentration of cracks, wounds, and sap-exuding areas on tree trunks near ground level, which provide suitable breeding and feeding sites for their larvae.

In our collection, the majority of the abundant Drosophilidae species belong to the group of mycophagous flies: *L. quinquemaculata*, *D. histrio*, *D. phalerata*, *D. testacea*, *D. transversa*, and *H. confusa* [50,51,52]. Most of these species showed a clear preference for the lower forest stratum, which is readily explained by its closer proximity to their primary food source—cap fungi. The only exception was *L. quinquemaculata*, which was significantly more abundant in the upper stratum of the forest interior. The larvae of this species developed in the fruiting bodies of various polypore fungi, particularly *Piptoporus betulinus* and *Fomitopsis pinicola* [53,54], which tend to occur more frequently at higher elevations on tree trunks.

Finally, *A. alboguttata* is one of the least studied species. However, there is some evidence regarding its ecology: larvae have been found under the bark of oak [45] and beech trees, and adults have been reared from the fungus *Daldinia concentrica*, which was growing on a burned birch trunk [55,56,57]. This fungus is a saprotrophic species that colonizes dead and decaying wood. In our study, *A. alboguttata* showed almost equal abundance across canopy levels at the forest edge (3 and 4 specimens, respectively), but in the forest interior, it clearly preferred the lower stratum (10 specimens in the lower traps vs. 3 in the canopy). These findings partly contradict previous reports suggesting that *A. alboguttata* prefers the upper tree stratum [28].

Thus, the bulk of drosophilids at the two study plots was represented by two ecological groups: xylosaprobionts and mycophagous species, with the latter dominating in terms of abundance. Of the 27 identified species, only 11 had a total abundance exceeding 10 specimens. These dominant species demonstrated distinct habitat preferences. Some species such as *G. distigma* and *D. transversa*, were more abundant at the forest edges, while others preferred the forest interior, including *A. alboguttata*, *L. quinquemaculata*, *P. semivirgo*, *D. obscura*, *D. phalerata*, *D. testacea*, *H. confusa*, and *S. rufifrons*. Species-specific vertical stratification was also evident: most species preferred the lower forest stratum (*A. alboguttata*, *P. semivirgo*, *D. histrio*, *D. obscura*, *D. phalerata*, *D. testacea*, *D. transversa*, *H. confusa*, and *S. rufifrons*), while the upper stratum was primarily inhabited by *G. distigma* and *L. quinquemaculata*.

However, a group of species in our collection was represented by only a single specimen, either at the forest edge or within the forest. Six species were collected exclusively at the forest edges: *S. coleoptrata*, *D. hydei*, *D. littoralis*, *D. melanogaster*, *H. trivittata*, and *S. pallida*. Despite their rarity, these species contributed to the overall higher species diversity of Drosophilidae at the forest edge. Most of them were recorded in plot 1. *S. coleoptrata* is a xylosaprobiont and a rather rare species, typically encountered as single individuals [42,43,54]. *D. hydei* and *D. melanogaster* are typical synanthropes that feed on rotting fruits, vegetables, and waste material [58,59,60,61,62]. These species were likely introduced from nearby human settlements, where stable populations are usually found. *D. littoralis* is a xylosaprobiont whose larvae develop under the bark of willow and alder; it is a rare species typically found near water bodies with willow vegetation [63,64]. *H. trivittata* is a mycophagous species associated almost exclusively with the fungi of the genus *Pleurotus* [65]. The larvae of *S. pallida* feed on decaying herbs, which are usually more abundant at forest edges [66]. Thus, the forest edge community appears to include a mixture of species typical of open habitats (including those introduced from anthropogenic environments) and forest-associated species.

Only three species were found exclusively within the forest: *A. albilabris*, *D. kuntzei*, and *D. repleta*. *A. albilabris* is a xylosaprobiont, similar to other members of the genus *Amiota* [67]. *D. kuntzei* is a typical mycophagous species that inhabits various types of fungi [28,50]. *D. repleta* is a synanthropic cosmopolitan species associated with human dwellings, where it feeds on decaying fruits, vegetables, and is often found in waste disposal sites [68], but the single specimen collected in the forest interior was most likely found due to accidental introduction, possibly by wind.

Can any of the aforementioned species be considered indicator species for the studied habitats? A comparison of habitat–species association assessments obtained through standard statistical methods and those derived using the Indicspecies R package shows a partial overlap between the resulting species lists. Both approaches confirm the role of *D. testacea* as an indicator species for the lower forest strata, and *L. quinquemaculata* as an indicator for the upper strata, but only in Plot 2. In addition, *S. rufifrons* appears to serve as an indicator of forest interior conditions (Table 4).

The observed discrepancies in species–habitat associations stem from the specific features of the IndVal metric used in the Indicspecies R package. This metric is calculated as the product of two components. The first is an estimate of the probability that a site where the species occurs belongs to a target group of sites, treating species presence or absence as a qualitative indicator. The second component is a probabilistic estimate of the species’ detectability at a site, which depends on its abundance. Due to this design, the role of low-abundance species becomes more prominent, especially if their presence is confined to specific clusters. In such cases, standard statistical methods often interpret abundance differences as random, while the multipatt function, which utilizes the IndVal metric, is more likely to identify such associations as significant. In our dataset, only 2 out of 11 drosophilid species identified using the χ^2^ method (18%) had a total abundance below 20 individuals (see Table 3), whereas among the 15 species identified as key indicators by Indicspecies, 5 species (33%) were low-abundance. Therefore, the most reliable habitat indicators are likely those species that appear in both sets of results obtained by the two statistical approaches.

Based on the study of Brazilian flies, it has been suggested that such interior-dwelling species are more sensitive to fluctuations in temperature and humidity than species living near forest boundaries [27]. Lewis [69] demonstrated that, on the leeward side of windbreaks, at a distance equal to the height of the windbreak, the density of gall midges (Cecidomyiidae), moth flies (Psychodidae), and fruit flies (Drosophilidae) was several times higher than in unprotected fields. Mendes et al. [16] described the seasonal patterns of drosophilid distribution and abundance at forest edges and inside forests. These studies suggest that Drosophilidae inhabiting forest edges are significantly influenced by a range of abiotic factors, including diurnal and seasonal variations in temperature, humidity, solar radiation, wind, and others. Moreover, the effects of microclimatic conditions may further shape insect responses in these habitats [70]. In addition, certain biotic factors—most notably the availability of food resources for both larvae and adults—play a key role in determining the distribution patterns of adult Drosophilidae.

## 5. Conclusions

Our study has several limitations that should be considered when interpreting the results. First, we used only baited traps with beer and sugar, which are not attractive to drosophilid species whose larvae develop in living plant tissues (e.g., some *Scaptomyza*) or in decaying herbaceous plants (e.g., *Lordiphosa*). Second, sampling was conducted only during June and July, whereas our previous study on the seasonal dynamics of drosophilid abundance in the same region (Mordovian State Nature Reserve) demonstrated sustained adult activity from May through October. Third, the total number of traps used was small, which may have affected the completeness of species representation. However, the same trapping protocol—two traps per site—was used in our earlier studies. In total, 27 species of Drosophilidae were identified in the forest ecosystems of central European Russia. Species richness was higher at forest edges in June and July, although the abundance of individual species was generally lower there compared to the forest interior. Nevertheless, the overall abundance of drosophilids was lower at forest edges than within the forest. Both abundance and species richness varied between the two study plots. Cumulative data showed that the Margalef index was higher at the forest edge than in the interior, while the Shannon and Simpson diversity indices exhibited only minor variation across different spatial and vertical positions in the traps. The greatest species diversity was recorded among xylosaprobiont and mycophagous drosophilids. All ecological groups were represented at the forest edge, whereas only four groups were found within the forest interior; notably, the phytosaprophagous species *S. pallida* was absent there. In general, drosophilid species richness and abundance were higher in the lower stratum both at the forest edge and inside the forest. Using the R package Indicspecies, we identified *G. distigma* as an indicator species of forest edges in both plots, and *S. rufifrons* as an indicator of forest interior in the lower stratum. Additionally, *D. testacea*, *D. phalerata*, and *P. semivirgo* were identified as indicator species for the lower stratum in both plots, while *L. quinquemaculata* was an indicator of the upper stratum in plot 2.

## Figures and Tables

**Table 1:** Abundance and species diversity of *Drosophilidae* collected using beer traps at forest edges and within forest interiors (summary data from two plots).

Species	Edge		Forest Interior	
	1.5 m	7.5 m	1.5 m	7.5 m
*Amiota albilabris* (Roth, 1860)	0	0	0	2
*Amiota alboguttata* (Wahlberg, 1839)	3	4	10	3
*Amiota rufescens* (Oldenberg, 1914)	2	2	2	3
*Amiota subtusradiata* Duda, 1934	0	1	0	0
*Gitona distigma* Meigen, 1830	6	8	3	5
*Leucophenga quinquemaculata* Strobl, 1893	3	5	9	24
*Phortica semivirgo* (Máca, 1977)	5	0	7	1
*Stegana coleoptrata* (Scopoli 1763)	1	0	0	0
*Stegana furta* (Linnaeus, 1767)	0	0	0	1
*Chymomyza amoena* (Loew, 1862)	1	0	1	0
*Chymomyza caudatula* Oldenberg, 1914	1	0	3	1
*Drosophila funebris* (Fabricius, 1787)	1	0	0	0
*Drosophila histrio* Meigen, 1830	2	0	30	4
*Drosophila hydei* Sturtevant, 1921	1	0	0	0
*Drosophila kuntzei* Duda, 1924	0	0	3	1
*Drosophila littoralis* Meigen, 1830	1	0	0	0
*Drosophila melanogaster* Meigen, 1830	1	0	0	0
*Drosophila obscura* Fallén, 1823	100	15	142	66
*Drosophila phalerata* Meigen, 1830	37	4	36	7
*Drosophila repleta* Wollaston, 1858	0	0	1	0
*Drosophila subobscura* Collin, 1936	1	0	2	0
*Drosophila testacea* von Roser, 1840	70	2	85	11
*Drosophila transversa* Fallén, 1823	68	9	20	5
*Hirtodrosophila confusa* (Staeger, 1844)	2	0	15	4
*Hirtodrosophila trivittata* (Strobl, 1893)	1	0	0	0
*Scaptodrosophila rufifrons* (Loew, 1873)	9	3	50	9
*Scaptomyza pallida* (Zetterstedt, 1847)	1	0	0	0
Total of individuals	317	53	419	147
Total of individuals (forest edge + interior)	370		566	
Number of species	22	10	17	16
Number of species (forest edge + interior)	23		19	
Margalef Index	3.65	2.27	2.65	3.01
Margalef Index (forest edge + interior)	3.72		2.84	
Shannon Index	1.89	2.04	2.03	1.94
Shannon Index (forest edge + interior)	1.98		2.08	
Simpson Index	0.79	0.84	0.81	0.76
Simpson Index (forest edge + interior)	0.80		0.81	

**Table 2:** Test for the homogeneity of distribution of the total abundance of individuals in accordance to the three factors—forest tier, forest depth, and plot.

Samples Grouped by Feature	G	d.f.	*p*-Value
Forest layer—1.5:7.5	124.81	26	7.10 × 10^−15^
Edge–forest interior	146.48	26	9.23 × 10^−19^
Plot 1–Plot 2	241.68	26	7.43 × 10^−37^

Plot 1—Selische, Plot 2—Sosnovka. G—significance of G-test value; d.f. –degrees of freedom.

**Table 3:** Influence of the factors “forest layer,” “forest depth,” and “plot” on the distribution of the abundance of individual species in the samples.

Species		Layer 1.5 m:7.5 m		Edge–Forest Interior		Plot 1–Plot 2	
	*n*	χ^2^	*p*	χ^2^	*p*	χ^2^	*p*
*A. albilabris*	2	7.38	**0.0066**	1.31	0.2524	4.23	0.0388
*A. alboguttata*	20	2.26	0.1327	0.18	0.6753	10.27	**0.0014**
*A. rufescens*	9	6.32	0.0119	0.09	0.7619	0.01	0.9284
*A. subtusradiata*	1	3.68	0.0549	1.53	0.2159	2.13	0.1442
*G. distigma*	22	19.08	**0.0000**	5.48	0.0193	1.89	0.1690
*L. quinquemaculata*	41	62.19	**0.0000**	7.19	**0.0073**	56.29	**0.0000**
*P. semivirgo*	11	1.47	0.2258	0.01	0.9368	0.48	0.4899
*S. coleoptrata*	1	0.27	0.6020	1.53	0.2159	0.47	0.4930
*S. furta*	1	3.68	0.0549	0.65	0.4185	2.13	0.1442
*C. amoena*	2	0.54	0.4605	0.09	0.7618	0.94	0.3321
*C. caudatula*	5	0.01	0.9404	0.8	0.3705	5.34	0.0209
*D. funebris*	1	0.27	0.6020	1.53	0.2159	0.47	0.4930
*D. histrio*	35	2.34	0.1258	18.08	**0.0000**	50.53	**0.0000**
*D. hydei*	1	0.27	0.6020	1.53	0.2159	2.13	0.1442
*D. kuntzei*	4	0.03	0.8590	2.63	0.1051	8.56	**0.0034**
*D. littoralis*	1	0.27	0.6020	1.53	0.2159	0.47	0.4930
*D. melanogaster*	1	0.27	0.6020	1.53	0.2159	0.47	0.4930
*D. obscura*	303	4.04	0.0444	3.18	0.0745	33.38	**0.0000**
*D. phalerata*	80	3.76	0.0525	3.32	0.0683	2.05	0.1525
*D. repleta*	1	0.27	0.6020	0.65	0.4185	0.47	0.4930
*D. subobscura*	3	0.82	0.3658	0.05	0.8260	1.67	0.1964
*D. testacea*	162	22.64	**0.0000**	0.95	0.3302	8.19	**0.0042**
*D. transversa*	82	3.98	0.0461	70.66	**0.0000**	15.65	**0.0001**
*H. confusa*	21	0.07	0.7931	8.09	**0.0045**	45.77	**0.0000**
*H. trivittata*	1	0.27	0.6020	1.53	0.2159	0.47	0.4930
*S. rufifrons*	67	0.91	0.3396	16.46	**0.0000**	3.76	0.0526
*S. pallida*	1	0.27	0.6020	1.53	0.2159	0.47	0.4930
B-H corr.	0.05	0.0074		0.0093		0.0148	

*n*—number of individuals of the given species. The bottom row of the table shows the Benjamini–Hochberg corrections for multiple comparisons. *p*-values lower than the respective thresholds are considered significant. Significant values are shown in bold.

**Table 4:** Significance estimates for indicator species characteristic of the surveyed plots.

Species	Layer 1.5:7.5	Forest Depth	Plots 1:2	*p*-Value
*D. testacea*	1.5	-	-	0.007
*D. phalerata*	1.5	-	-	0.0264
*P. semivirgo*	1.5	-	-	0.0284
*D. kuntzei*	-	forest	Plot 2	0.0360
*H. confusa*	-	forest	Plot 2	0.0360
*C. caudatula*	-	forest	Plot 2	0.0450
*L. quinquemaculata*	-	-	Plot 2	0.0080
*H. confusa*	-	-	Plot 2	0.0250
*L. quinquemaculata*	7.5	-	Plot 2	0.0360
*G. distigma*	-	edge	Plot 2	0.0370
*S. rufifrons*	1.5	forest	-	0.0430

Samples are grouped according to one or two listed characteristics. Two characteristics are applied when a sampling point characteristic is restricted within another category (e.g., trap position relative to the forest edge).

**Table 5:** Combinations of potential indicator species for forest layers and sampling plots.

Pairs and Triplets of Species	Layer 1.5:7.5	Sites1:2	*p*-Value
*D. obscura + D. phalerata + D. testacea*	-	site 1	0.0278
*D. phalerata + D. testacea*	-	site 1	0.0278
*D. testacea + D. transversa*	1.5	-	0.0149
*D. obscura + D. transversa*	1.5	-	0.0353
*D. testacea + D. obscura*	1.5	-	0.0097
*D. testacea + P. semivirgo*	1.5	-	0.0174
*D. phalerata + D. testacea*	1.5	-	0.0017
*D. phalerata + D. obscura*	1.5	-	0.0193
*D. phalerata + D. transversa*	1.5	-	0.0353
*D.phalerata + L. quinquemaculata*	1.5	-	0.0433
*P. semivirgo + D. obscura*	1.5	-	0.0307
*P. semivirgo + D. phalerata*	1.5	-	0.0307
*P. semivirgo + D. transversa*	1.5	-	0.0307
*P. semivirgo + S. rufifrons*	1.5	-	0.0307
*D. testacea + S. rufifrons*	1.5	-	0.0449
*D. phalerata + S. rufifrons*	1.5	-	0.0430

## Data Availability

The original contributions presented in this study are included in the article/Supplementary Materials. Further inquiries can be directed to the corresponding author.

## Supplementary Materials

The following supporting information can be downloaded at https://www.mdpi.com/article/10.3390/insects16080762/s1, Table S1: Descriptive statistics for the distribution of species abundance at collection sites.
