# Supplementary material for: Edge Effects on the Spatial Distribution and Diversity of Drosophilidae (Diptera) Assemblages in Deciduous Forests of Central European Russia
Source: Insects. 2025 Jul 24;16(8):762. doi: 10.3390/insects16080762 (PMC12386307; doi:10.3390/insects16080762)
Supplement: Supplementary file 1 [file insects-16-00762-s001.zip › insects-3723383-supplementary.pdf]

Supplementary materials

Table S1. Descriptive statistics for the distribution of species abundance at collection sites.

	Valid N	M	Med	Min	Max	Low. Q	Up. Q	Std. Dev.	Skewness	Std. Err. Sk.	Kurtosis	Std. Err. K
Sel_forest_1.5	27	8.67	0	0	101	0	4	22.60	3.4	0.45	11.8	0.87
Sel_forest_7.5	27	2.41	0	0	49	0	1	9.43	5.0	0.45	25.5	0.87
Sel_edge_1.5	27	11.15	1	0	97	0	5	25.03	2.6	0.45	5.7	0.87
Sel_edge_7.5	27	1.37	0	0	12	0	1	3.01	2.6	0.45	6.4	0.87
Sos_forest_1.5	27	6.85	2	0	41	0	11	10.65	1.8	0.45	3.0	0.87
Sos_forest_7.5	27	3.04	1	0	24	0	4	5.67	2.8	0.45	7.7	0.87
Sos_edge_1.5	27	0.59	0	0	4	0	1	1.05	2.0	0.45	3.8	0.87
Sos_edge_7.5	27	0.59	0	0	5	0	1	1.22	2.4	0.45	6.0	0.87

Valid N – number of species recorded at the collection site; M – mean species abundance; Med – median species abundance; Min – minimum abundance per species; Max – maximum abundance per species; Low. Q – lower quartile; Up. Q – upper quartile; Std. Dev. – standard deviation; Skewness – asymmetry of the distribution; Std. Err. Sk – standard error of skewness; Kurtosis – excess of the distribution; Std. Err. K – standard error of kurtosis. In all cases, the distributions significantly deviate from normality (Kolmogorov -Smirnov $d<0.427$, $p<0.01$; Lilliefors $p<0.01$).
